# Threat-Modulation of Executive Functions—A Novel Biomarker of Depression?

**DOI:** 10.3389/fpsyt.2021.670974

**Published:** 2021-07-02

**Authors:** Jari Peräkylä, Kaija Järventausta, Piia Haapaniemi, Joan A. Camprodon, Kaisa M. Hartikainen

**Affiliations:** ^1^Behavioral Neurology Research Unit, Tampere University Hospital, Tampere, Finland; ^2^Faculty of Medicine and Health Technology, Tampere University, Tampere, Finland; ^3^Department of Psychiatry, Tampere University Hospital, Tampere, Finland; ^4^Department of Psychiatry, Harvard Medical School, Massachusetts General Hospital, Boston, MA, United States

**Keywords:** ECT, MDD, depression, executive function, threat, biomarker, emotion, attention

## Abstract

**Background:** Alterations in executive functions, emotion regulation, and their interaction are common concomitants of depression. Executive dysfunction frequently lingers after treatment, has adverse effects on daily life, and predisposes to recurrence of depression. Yet, sensitive measures of executive function for reliable assessment of cognitive outcomes are still lacking in clinical practice. To better understand the impact of depression and its most effective treatment, electroconvulsive therapy (ECT), on cognition, we assessed executive functions pre- and post-ECT and whether objective measures reflecting alterations in emotion–executive function interaction correlate with depression severity or with cognitive outcome.

**Methods:** Executive functions were assessed in 21 patients with major depressive disorder (MDD) before and after ECT using subjective measures from the Behavior Rating Inventory of Executive Function—Adult version (BRIEF-A) and objective cognitive performance measures derived from computer-based test of executive function, Executive Reaction Time (RT) Test. In addition, we created novel indices reflecting emotional modulation of cognitive performance by subtracting different performance measures in the context of neutral distractors from those in the context of threat-related distractors. We correlated these indices with Beck Depression Inventory (BDI) and BRIEF-A scores.

**Results:** Depression was significantly alleviated, and executive functions improved post-ECT, as seen in reduced BDI scores, BRIEF-A scores, and number of errors in Executive RT Test. Pre-ECT BDI scores correlated with threat modulation of RT (tmRT) and threat modulation of working memory (tmWM). Post-ECT tmRT correlated with several Behavioral Regulation scales and tmWM with several Metacognition scales of BRIEF-A.

**Conclusion:** While caution is warranted, results from both subjective and objective measures suggest that ECT significantly improves executive functions and emotion regulation along with alleviation of depression. Novel indices derived from threat modulation of executive function and working memory show promise as objective biomarkers of depression severity pre-ECT and cognitive outcome post-ECT with potential for guiding depression treatments.

## Introduction

The diagnosis of major depressive disorder (MDD) is primarily based on clinical examination and subjective evaluation of depressive symptoms with objective tests of depression lacking in the current clinical practice. Likewise, reliable and easy-to-obtain measures for cognitive outcomes of depression treatments are still missing considering the limitations of traditional neuropsychological assessments. Identifying novel biomarkers of depression severity and cognitive outcome will have broad implications in developing diagnostic tools, treatment selection, and optimizing neuromodulation treatments. Furthermore, discovery of novel biomarkers of depression will have wide impact not only on clinical practice but also on scientific endeavors aiming to gain better understanding of depression and its treatments.

The most effective treatment of pharmacoresistant depression is electroconvulsive therapy (ECT) (1, 2). There are many other invasive and non-invasive neuromodulation treatments for depression (3) with vast parameter space that need to be optimized to provide best possible treatment outcome with minimal side effects. The selection of neuromodulation parameters is based mainly on previous experience and clinical evaluation at follow-up visit. Thus, optimizing stimulation parameters online is a challenge and calls for biomarkers that reflect the immediate impact of neuromodulation on cognitive and affective brain functions of the treated individual.

MDD is associated with cognitive and affective dysfunction, specifically executive dysfunction and emotion dysregulation (4–9). Consequently, it is feasible that biomarkers reflecting functioning of emotion regulation and cognitive control circuits might reflect severity of depression. Emotion regulation and cognitive control share the same underlying neural networks (10). On the other hand, altered functional connectivity of cognitive control networks contributes to both executive dysfunction and depression (11). Current antidepressant therapies are primarily effective for emotional and autonomic symptoms of MDD but not for cognitive deficits. Even when depression is in remission, executive dysfunction may persist (4, 12–14). With adaptive emotion regulation strategies relying heavily on executive functions (15, 16), executive dysfunction in MDD contributes to an increased risk of relapse (17). In addition to increased relapse risk, executive dysfunction impairs daily life functioning. Thus, it is of outmost importance to assess and optimize the impact of depression treatments on executive functions.

Executive dysfunction may not always be depicted with conventional neuropsychological tests, even when interfering with everyday life (18–20). Furthermore, substantial learning effect limits reliable use of standardized neuropsychological tests in the assessment of the impact of an intervention, such as neuromodulation, on executive functions (21). Additionally, neuropsychological tests are performed in structured and emotionally neutral surroundings, unlike unstructured and emotionally burdening everyday life situations, which require far greater extent of cognitive control (22, 23). To that end, adding an emotional distractor, such as threat-related stimulus, is called for when mimicking everyday requirements for cognitive control.

It is crucial for survival that individuals pay attention to threat-related stimuli and allocate adequate cognitive control resources to minimize the threat, even when busy with a task engaging attention and executive functions (24, 25). To facilitate this, threat-related stimuli have prioritized access to attentional and executive function resources (24–27). When threat-related stimuli compete for the same attentional and cognitive control resources as the ongoing task, task performance may be compromised (26, 28–30). Healthy individuals with well-functioning emotional and cognitive control circuits are capable of disengaging attention from negative emotional event and suppressing undue emotional responding when adequate assessment suggests no threat. This is in contrast to individuals with depression who have reduced capability to do so (31), leading to exaggerated and prolonged impact of intervening threat-related stimuli on task performance. Greater and prolonged allocation of attention to negative emotional stimuli is a key emotion regulation problem in depression referred to as a negativity bias (32–35). Negativity bias is thought to be a risk factor of depression and contribute to development and maintenance of depression and anxiety (36). Executive functions are especially vulnerable in depression. On the other hand, executive functions are needed for controlling undue impact of intervening threat-related stimuli on task performance. To that end, the impact of threat on tasks requiring executive function may have some benefit as a depression biomarker over those based merely on attentional bias.

In this study, we studied whether the threat-related distractors impact task performance in tasks requiring multiple executive functions simultaneously, i.e., threat modulation of task performance. We assumed that patients with more severe depression would have greater impact of threat-related distractor on task performance. Threat-related stimuli may impact cognitive performance in multiple ways either impairing or improving task performance. Whether improvement or decrement is observed depends on several emotion-, individual-, and task-related factors. Negative emotional task-irrelevant stimuli may compete for the same attentional and cognitive control resources as task-relevant stimuli, leading to task interference, such as impaired response inhibition, impaired attention to global level visual features, or impaired attention to left visual field targets (26, 28–30). On the other hand, threat-related stimuli may increase arousal, improving performance (37, 38). Whether emotional arousal leads to improvement or decrement in cognitive performance is determined by task difficulty and baseline level of arousal in each individual according to inverted U-curve (39, 40).

In this study, we investigated the effect of ECT on executive functions and emotional control. We hypothesized that ECT treatment will improve executive functions and, along with improved executive functions, enhance emotional control. To assess executive functions and emotional control, we used both objective and subjective measures, i.e., an experimental computer-based test of executive functions, Executive Reaction Time (RT) Test (48), and a clinically validated questionnaire, Behavior Rating Inventory of Executive Function—Adult Version (BRIEF-A), a standardized measure of executive functions and self-regulation in daily life based on self-report (41).

With aims to discover novel biomarkers of depression, we created novel *threat modulation indices* for RT (tmRT) and working memory (tmWM) by subtracting performance measures in the context of neutral distractors from corresponding performance measures in the context of threat-related distractors. These performance measures were derived from the Executive RT Test, an experimental computer-based test objectively assessing efficiency of different executive functions including working memory in the context of threat-related distractors (48). Threat modulation indices provided a numeric value for the extent of performance modulation by threat. Finally, we correlated these indices (tmRT and tmWM) with Beck Depression Inventory (BDI) (42) and Mongomery–Asberg Depression Scale (MADRS) (43) scores, as well as with BRIEF-A (44) scores, to assess their potential as biomarkers for depression severity and cognitive outcomes of depression treatments.

## Subjects and Methods

### Subjects

Thirty subjects treated for pharmacoresistant MDD with ECT at Tampere University Hospital were recruited for the study. The study was approved by the Tampere University Hospital Ethical board, and participants gave their written consent according to the Declaration of Helsinki. Inclusion criteria were pharmacoresistant MDD and 18–85 years of age. Exclusion criteria were other major psychiatric or neurological conditions, non-correctable vision problem, and alcohol or drug abuse.

The final study group consisted of 17 subjects (8 female and 9 male). Thirteen subjects did not complete the study. Some subjects withdrew from the study at an early phase due to lack of sufficient mental energy to complete the experimental task and some due to interrupted ECT treatment. Fourteen participants had an International Statistical Classification of Diseases and Related Health Problems 10th revision (ICD-10) diagnosis code F33.2 (recurrent severe major depressive disorder without psychotic features) and three F32.2 (single episode major depressive disorder without psychotic features). In the final study group, the mean age of the subjects was 37.4 (SD, 14.1; range, 20–59) years.

### Study Design

Subjects participated in two testing sessions: one just prior to the first ECT session (pre-ECT) and another after the completion of the course of ECT treatment period (post-ECT). Subjects' treatment period was 3–4 weeks, three sessions per week, and an average number of sessions was 10.9 (SD, 4.0). ECT session count varied between 4 and 21 and was guided by clinical status of the patient. Post-ECT testing was done 4–8 days after the completion of the treatment period. All patients continued their prescribed medications during ECT, and their medications were not changed during the study.

### ECT

ECT was administered in Tampere University Hospital Psychiatric Ward following hospital ECT protocol. ECT was applied bifrontally using a MECTA Spectrum 5000Q ECT device (MECTA Corporation, Lake Oswego, OR, USA). Stimulation mode was a brief pulse (0.5 ms) constant current stimulation, and seizure threshold was titrated during the first session. In subsequent sessions, treatment was given at the level of 1.5 × titrated threshold. ECT energy was 81 mC for 3 patients, 157 mC for 1 patient, 162–163 mC for 11 patients, and 324–1,152 mC for 2 patients.

### Assessment of Depression and Executive Functions

Subjects' depression severity was assessed with Beck Depression Inventory II (BDI-II) and Montgomery–Åsberg Depression Rating Scale (MADRS) questionnaires (42, 43). Executive functions were assessed using Executive RT Test (48) and BRIEF-A (41).

BRIEF-A questionnaire is designed for the subjective evaluation of executive functions in everyday life. It is composed of eight rating scales assessing distinct aspects of executive functions (self-monitoring, planning and organizing, organization of materials, working memory, initiation of tasks, shifting, inhibition, and emotional control) and three composite indices (metacognition index, behavioral regulation index, and global executive composite) combining individual indices into higher-level composite scores.

**Figure 1:**
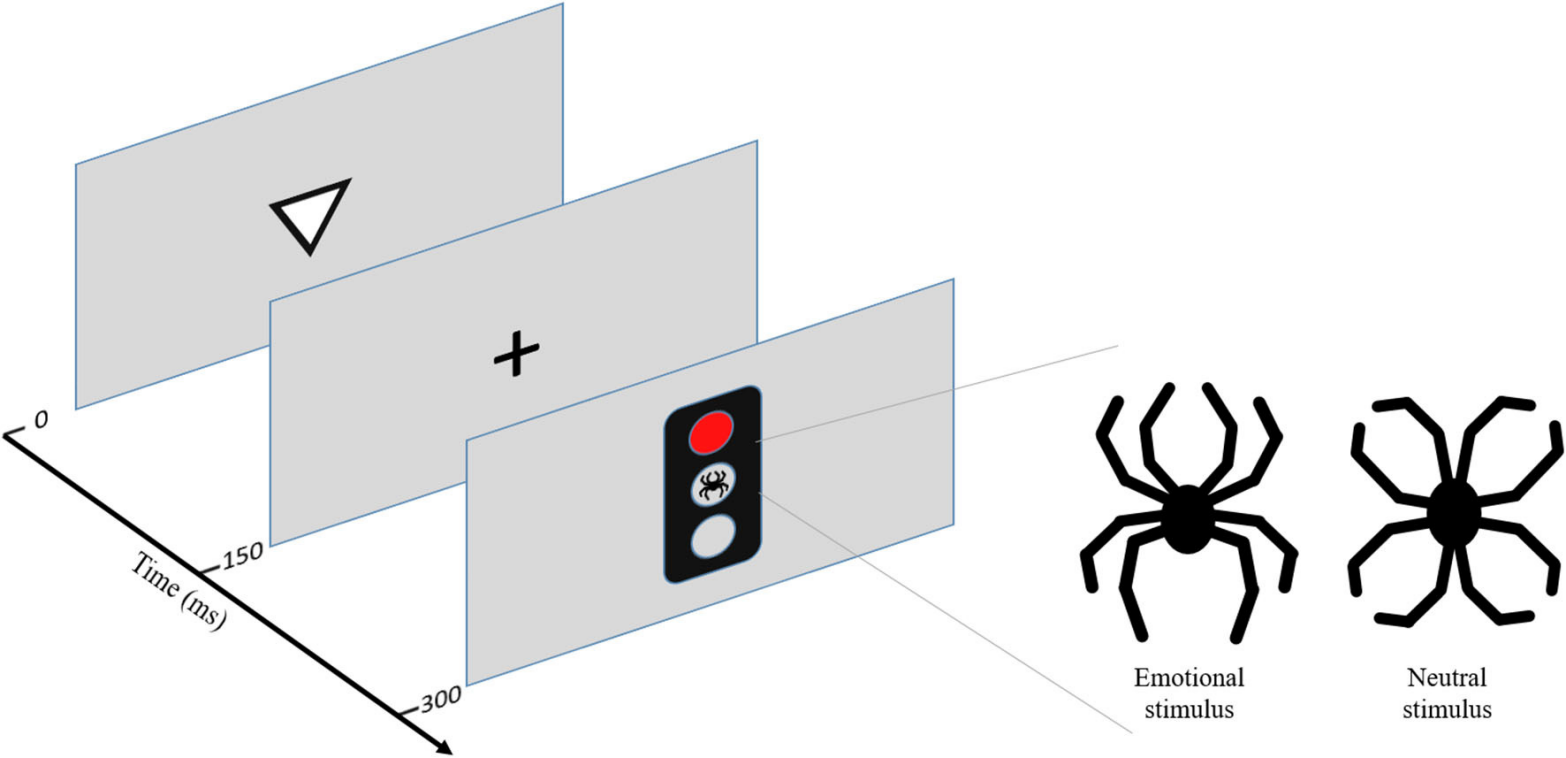
Executive Reaction Time (RT) Test. Executive RT Test is a computer-based experimental Go/NoGo test with threat-related distractors (48). Executive RT Test has been designed to engage multiple executive functions (working memory, inhibition, attention, and shifting) simultaneously to mimic real life requirements for executive functions. Additionally, Executive RT Test has an emotional component in the form of a threat-related distractor to simulate real life situations where executive functions are needed in emotionally charged situations. The task of the subject is to indicate the orientation of the triangle by pressing one of the two response buttons as fast as possible when a Go signal is presented and withhold from responding when a NoGo signal is presented. The color of the traffic light indicates a Go- or a NoGo trial, with green light indicating a Go trial and red light indicating a NoGo trial in half of the blocks and the other way round in the other half of the blocks. The rule for responding changes every two blocks. There are 64 trials in 8 blocks. The outcomes of the test are RTs and error rates. Different error modalities are assumed to reflect different aspects of executive functions; incorrect responses reflect lapses in working memory, missed responses lapses in attention, and commission errors failures in response inhibition. Individual error types can be summed up to reflect overall performance. RTs reflect overall executive functions and have been shown to correlate with subjective evaluations of executive functions in daily life in healthy subjects (49). The test has been previously used to detect subtle changes in executive functions, both impairments and improvements, due to neuromodulation (46, 50, 51), mild traumatic brain injury (MTBI) (48), and major cardiac operations (52). In addition, the test is sensitive in depicting alterations in emotion–attention interaction due to MTBI (53), focal brain injury (54), and neuromodulation (45–47, 55). Executive RT Test has been shown to be reliable in repeated testing and reaction time to reflect executive function in daily life in healthy subjects (49). Figure adapted from (51).

For objective assessment of executive functions, performance measures of the computer-based test of executive functions, Executive RT Test, were used. In addition, the Executive RT Test allows for the assessment of emotion–attention and emotion–executive function interaction and alterations due to neuromodulation (45–47) in them. See Figure 1 for the description of the Executive RT Test.

### Threat-Modulation Indices

Comparing performance in the context of a threat-related distractor, a line drawing of a spider, to performance in the context of an emotionally neutral control figure resembling a flower, composed of the exact same line elements as the spider, allows for assessing the impact of mere threat on attention and executive functions with the impact of a visual distractor on performance controlled for. We calculated a *threat modulation index* for each subject by subtracting a cognitive measure, either reaction time or number of incorrect responses, in the context of neutral distractor from the same measure in the context of threat-related distractor, thus isolating the impact of a threat on RT (tmRT) reflecting the impact of threat on executive functions in general and on working memory (tmWM), correspondingly.

### Statistical Analysis

BDI and MADRS scores were analyzed using a paired *t*-test and BRIEF-A scores and threat modulation indices with Wilcoxon signed-rank test. Reaction times in the Executive RT Test were analyzed with repeated measures ANOVA using Distractor Valence (Emotional, Neutral) and Test (pre-ECT, post-ECT) as factors. Errors in Executive RT Test were analyzed with generalized binary logistic regression using Distractor Valence (Emotional, Neutral) and Test (pre-ECT, post-ECT) as fixed effect predictors and Subject as random effect predictor. Each error type (incorrect responses, missing responses, commission errors, and total errors) had its own model, and before analysis, trial outcomes were dichotomized so that total errors outcome was “error” or “correct,” incorrect responses outcome “incorrect” or “other,” missing responses “miss” or “other,” and commission errors “commission error” or “correct.” If significant interactions were found, data were stratified into respective groups, and groups were analyzed separately.

Correlation analyses between subjective (BRIEF-A) and objective (Executive RT Test) measures of executive functions, depression severity (BDI, MADRS), and threat modulation indices were done using Spearman rank-order correlation. As correlation analysis is sensitive to outliers (56), possible outlier data points were identified and removed from the analysis using Cook's distance with cutoff value of 4/n and Mahalanobis distance with cutoff value of 5.99. Significances were adjusted for multiple comparison with the Benjamini and Hochberg procedure (57, 58). The normality assumption of the data was confirmed with QQ plots and Shapiro–Wilk tests. Statistical analysis was conducted with R: A language and environment for statistical computing, version 3.6.2. (59) with add-on packages “lme4” version 1.1-21 (60) (regression analysis) and “ez” version 4.4-0 (61) (repeated measures ANOVA).

## Results

### The Effect of ECT on Depression

ECT alleviated depression significantly. Subjects' pre-ECT mean BDI score was 35.2 (SD = 7.7) and MADRS score of 36.4 (6.9), both indicating severe depression. Post-ECT mean BDI score was 17.8 (11.2), and MADRS score was 11.9 (10.5), both indicating mild depression. The changes in MADRS and BDI scores were statistically significant, MADRS *p* < 0.001 and BDI *p* < 0.001, and highly correlated, *r* = 0.75, *p* < 0.001.

### The Effect of ECT on Executive Functions

**Figure 2:**
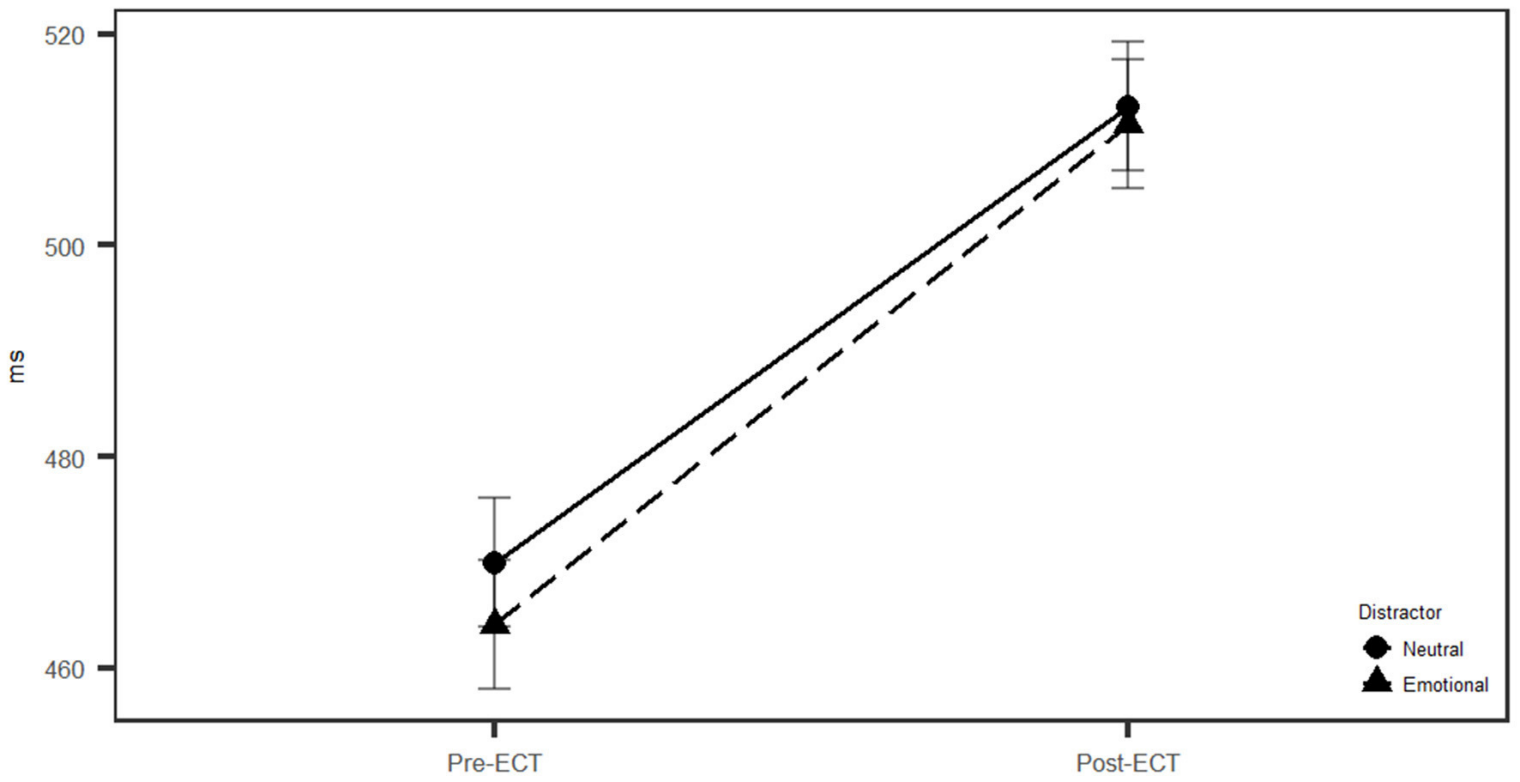
Reaction times in the context of threat related and neutral distractor pre- and post-electroconvulsive therapy (post-ECT). Pre-electroconvulsive therapy (ECT) subjects had faster reaction time, and emotional distractor speeded up their reaction times (RTs) compared to neutral distractor (*p* = 0.006).

RTs in the Executive RT Test were significantly slower post-ECT (512 ms, SD = 127 ms) than pre-ECT [467 (116) ms, *F*_(1, 16)_ = 5.80, *p* = 0.028, $\eta_{\text{G}}^{2}$ = 0.04] (Table 1). Threat-related distractors were associated with faster RTs pre-ECT but not post-ECT [Test × Emotion interaction, *F*_(1, 16)_ = 4.64, *p* = 0.047, $\eta_{\text{G}}^{2}$ = 0.00; threat pre-ECT, 464 (116); neutral pre-ECT, 470 (115) ms, *F*_(1, 16)_ = 9.80, *p* = 0.006, $\eta_{\text{G}}^{2}$ = 0.00; threat post-ECT, 511 ± 126; neutral post-ECT, 513 (130) ms, *F*_(1, 16)_ = 0.02, *p* = 0.898, $\eta_{\text{G}}^{2}$ = 0.00] (Figure 2).

**Table 1:** Performance in the Executive RT Test in the context of threat-related and neutral distractor pre- and post-ECT.

**Test**	**Distractor**	**Mean RT (ms)**	**Median total Errors (%, IQR)**	**Median incorrect responses (%, IQR)**	**Median missing responses (%,IQR)**	**Median commission errors (%, IQR)**
Pre-ECT	Overall	467 (114)	2.3 (3.1)	1.0 (3.0)	0.5 (1.0)	0.3 (1.0)
	Neutral	470 (115)	2.1 (3.1)	1.0 (2.1)	0.5 (1.0)	0.5 (1.0)
	Threat	464 (116)	2.6 (3.1)	1.0 (3.1)	0.5 (1.0)	0.0 (1.0)
Post-ECT	Overall	512 (126)	1.0 (2.3)	0.5 (2.1)	0.0 (0.4)	0.0 (0.0)
	Neutral	513 (130)	1.0 (2.1)	0.5 (2.1)	0.0 (0.5)	0.0 (0.0)
	Threat	511 (126)	1.0 (2.1)	0.0 (2.1)	0.0 (0.0)	0.0 (0.5)

*IQR, interquartile range*.

**Figure 3:**
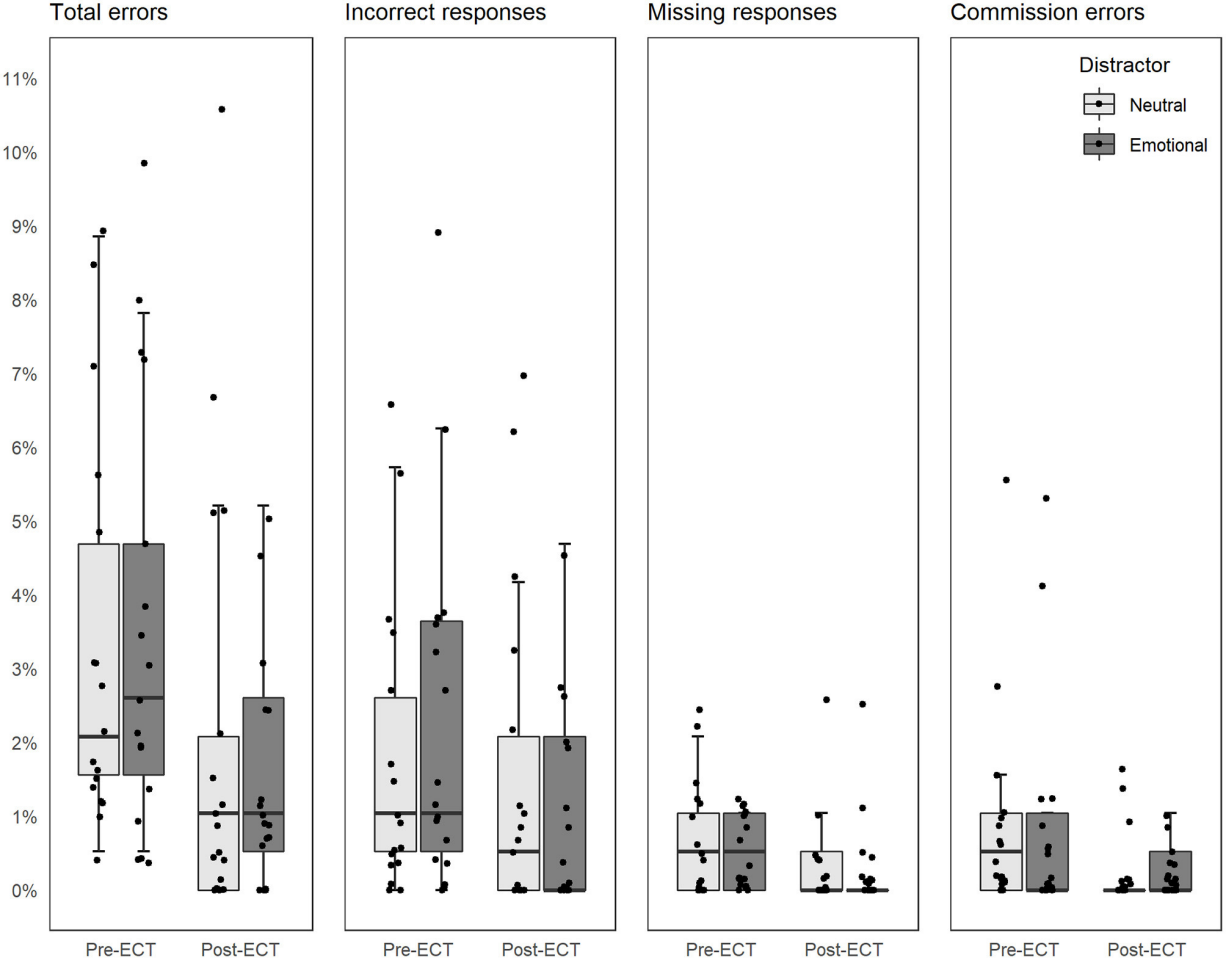
Error rates in the context of threat related and neutral distractor pre- and post-ECT. All figures share the Y-axis on the left. Dots indicate individual subjects and have been jittered to separate individual subjects.

The analysis of total errors, missing responses, and commission errors revealed that subjects were 37% less probable to make an error in general [total errors, odds ratio (OR) = 0.63, CI = 0.46–0.86], more specifically, 53% less probable to miss responding in Go condition (missing responses, OR = 0.47, CI = 0.23–0.98) and 72% less probable to fail in withholding a response in NoGo condition (commission error, OR = 0.28, CI = 0.13–0.60) post-ECT (Figure 3).

The analysis of incorrect responses resulted in Test × Distractor Valence interaction. When data were stratified based on distractor valence, subjects were 59% less probable to respond incorrectly post-ECT in the context of a threat-related distractor compared to pre-ECT (OR = 0.41, CI = 0.27–0.63). There was no difference in the context of the neutral distractor (OR = 0.87, CI = 0.59–1.28). When data were stratified based on test, there were no difference between distractors pre-ECT (OR = 1.28, CI = 0.90–1.83), but post-ECT subjects were 39% less likely to respond incorrectly in the context of threat-related distractor compared to neutral distractor (OR = 0.61, CI = 0.39–0.95).

When pre- and post-ECT BRIEF-A T-scores (Table 2) were analyzed, all summary indices improved significantly: general executive composite (GEC) improved from 72.3 to 66.7, *p* = 0.016; emotional control index (BRI) from 66.1 to 61.6, *p* = 0.043; and problem solving and working memory index (MI) from 74.4 to 68.6, *p* = 0.046. Out of the individual scales, Inhibitory control (from 63.5 to 59.2, *p* = 0.015), Initiation of tasks (from 80.6 to 69.5, *p* = 0.001), Flexibility to shift from one task to another (from 69.8. to 64.7, *p* = 0.014), and Monitoring of one's own task execution performance (from 72.2 to 65.8, *p* = 0.049) were significantly improved post-ECT. When corrected for multiple comparison, adjusted *p*-values—*p*(adj)—for GEC and Inhibit, Shift, and Initiate scales remained significant, while BRI, MI, and Task Monitoring approached significance. All scales except

**Table 2:** Behavior rating inventory of executive function—adult version (BRIEF-A) T-scores pre- and post-ECT.

**Scale**	**Pre-ECT**	**Post-ECT**	**Difference**	***p*-value**	**p(adj)**
Global Executive Composite (GEC)	72.3 (12.5)	66.7 (13.4)	−5.6 (8.3)	**0.016**	**0.047**
Behavioral Regulation Index (BRI)	66.1 (11.0)	61.6 (14.1)	−4.5 (8.5)	**0.043**	0.085
Inhibit	63.5 (12.8)	59.2 (13.1)	−4.3 (6.5)	**0.015**	**0.047**
Shift	69.8 (12.6)	64.7 (13.2)	−5.1 (6.5)	**0.014**	**0.047**
Emotional control	63.4 (11.4)	58.4 (14.7)	−5.0 (11.4)	0.070	0.105
Self-monitoring	55.7 (12.2)	57.2 (13.5)	1.5 (10.4)	0.726	0.726
Metacognition Index (MI)	74.4 (13.2)	68.6 (12.3)	−5.8 (10.3)	**0.046**	0.085
Initiate	80.6 (8.7)	69.5 (9.5)	−11.1 (8.2)	**0.001**	**0.015**
Working memory	73.9 (12.4)	67.9 (15.4)	−6.0 (14.0)	0.139	0.185
Plan/organize	68.0 (14.6)	65.9 (11.5)	−2.1 (11.9)	0.414	0.497
Task monitoring	72.2 (14.0)	65.8 (13.1)	−6.4 (11.8)	**0.049**	0.085
Organization of materials	61.9 (12.8)	60.8 (12.1)	−1.1 (8.2)	0.726	0.726

*Format: rho (p/p-adj). Bold values indicate p < 0.05*.

Self-Monitoring had lower post-ECT score indicating improved functioning in daily life.

There were no systematic correlations between direct outcome measures of Executive RT Test and BRIEF-A before or after ECT.

### The Effect of ECT on Emotion Regulation

There was a statistically significant difference in threat modulation index for incorrect responses. Threat modulation of working memory (tmWM) was +0.9% points pre-ECT, indicating that threat-related distractor impaired the working memory performance pre-ECT, and −1.1% points post-ECT, indicating that threat-related distractor improved working memory performance post-ECT. The difference was statistically significant, *V* = 91.5, *p* = 0.014. The difference between pre- and post-ECT tmRT had a trend toward significance, −5.8 ms pre-ECT and −1.7 ms post-ECT, *V* = 40, *p* = 0.089.

### Correlation of Threat Modulation Indices With MADRS, BDI, and BRIEF-A

**Figure 4:**
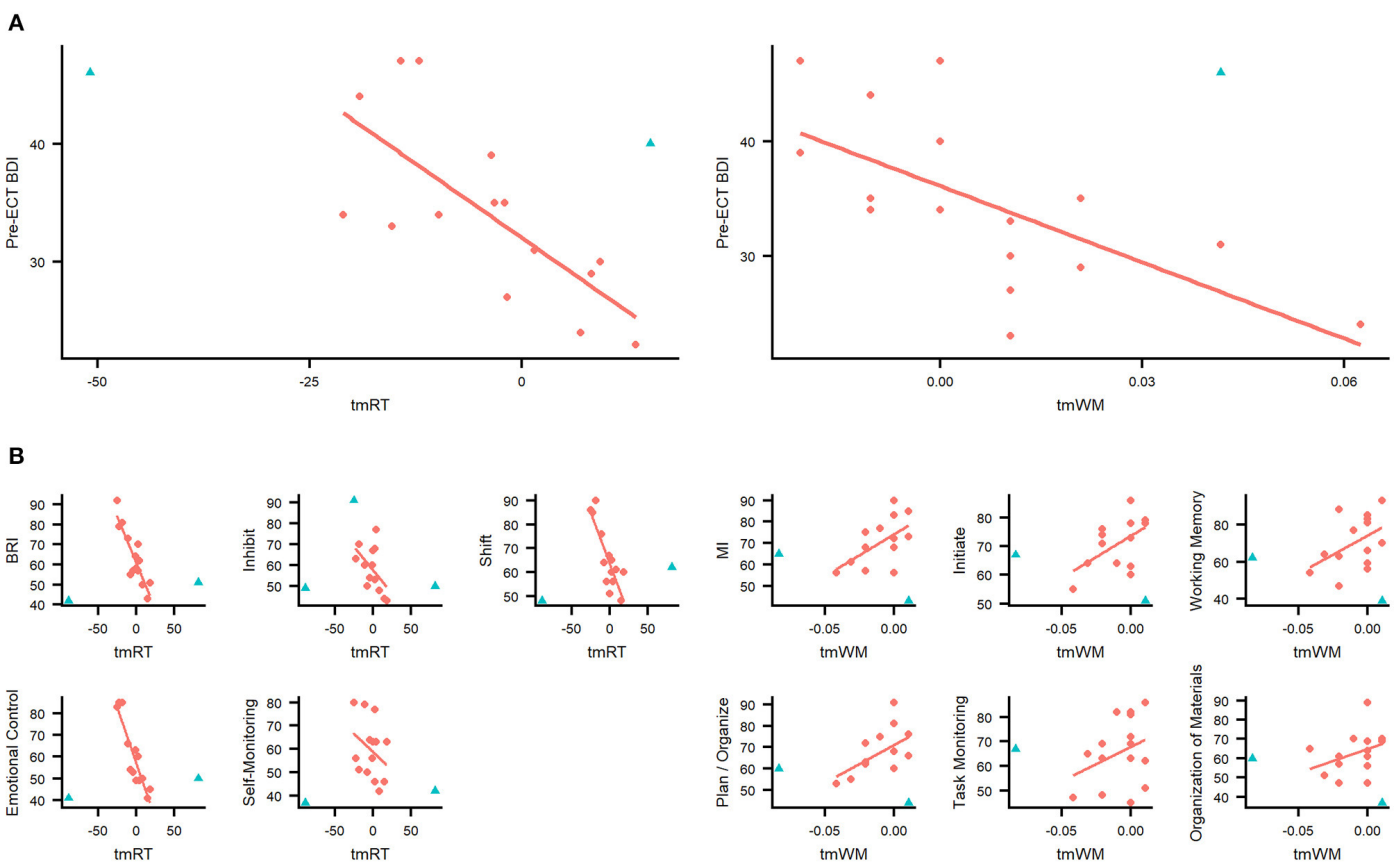
**(A)** Novel threat modulation indices reflect depression severity pre-ECT. Pre-ECT threat modulation index correlations with Beck Depression Inventory (BDI) score for incorrect responses (tmWM) and reaction time (tmRT). Both indices correlated significantly (Spearman correlation) with BDI score pre-ECT. **(B)** Novel threat modulation indices reflect executive functioning and emotion regulation in daily life post-ECT. Post-ECT threat modulation index correlations with Behavior Rating Inventory of Executive Function—Adult version (BRIEF-A) scales and indices. tmRT correlated significantly with BRI index and Inhibit, Shift, Emotional control and Self-monitoring scales. All of these scales are part of the BRI index. tmWM correlated with the MI scale and Initiate, Working memory, Plan and organize, and Self-monitoring scales. All of these scales are part of the MI index. Red dot, subjects included in correlation analysis; blue triangle, subject is an outlier and excluded from correlation analysis. Outliers were identified using Cook's and Mahalanobis distances.

Correlation analysis of threat modulation indices and BDI score resulted in two significant sets of correlations. Pre-ECT tmRT and tmWM correlated with pre-ECT BDI score [ρ(tmRT) = −0.75, p(tmRT) = 0.001, p(adj)(tmRT) = 0.010; ρ(tmWM) = −0.71, p(tmWM) = 0.002, p(adj)(tmWM) = 0.012 (see Table 3A and Figure 4A)]. These correlations were not present post-ECT, and there were no correlations between threat modulation indices and MADRS scores.

**Table 3A:** Significant correlations between threat modulation indices and BDI scores pre-ECT.

**Variable**	**Pre-ECT BDI**	**Pre-ECT MADRS**
tmRT	−0.73 (0.002/0.012)	−0.14 (0.608/0.839)
tmWM	−0.71 (0.002/0.012)	−0.30 (0.252/0.605)

*Threat modulation indices for RT (tmRT) and incorrect errors (tmWM) correlate with BDI scores. Format: rho (p/p-adj)*.

Post-ECT tmRT correlated with several behavior regulation-related BRIEF-A scales (BRI, Inhibit, Shift, and Emotional control) and tmWM with several metacognition-related BRIEF-A indices (MI, Initiate, Working memory, and Plan and organize). tmRT remained significant even after correction for multiple comparison (see Table 3B and Figure 4B). There were no similar correlations pre-ECT.

**Table 3B:** Significant correlations between threat modulation indices and BRIEF-A questionnaire post-ECT.

**Variable**	**Global executive composite (GEC)**	**Behavioral regulation index (BRI)**	**Inhibit**	**Shift**	**Emotional control**	**Self-monitoring**
tmRT	−0.53 (0.052/0.234)	**−0.78 (0.001**/**0.022)**	−0.45 (0.124/0.321)	**−0.73 (0.003**/**0.046)**	**−0.86 (0.000**/**0.005)**	−0.35 (0.227/0.375)
tmWM	**0.72 (0.004**/**0.046)**	0.44 (0.112/0.321)	0.35 (0.217/0.375)	0.35 (0.196/0.361)	0.41 (0.127/0.321)	0.32 (0.244/0.378)
**Variable**	**Metacognition index (MI)**	**Initiate**	**Working memory**	**Plan/organize**	**Task monitoring**	**Organization of materials**
tmRT	−0.39 (0.172/0.345)	−0.34 (0.241/0.378)	−0.30 (0.302/0.415)	−0.28 (0.327/0.424)	−0.32 (0.265/0.398)	−0.45 (0.123/0.321)
tmWM	**0.55 (0.042**/**0.234)**	**0.54 (0.048**/**0.234)**	0.51 (0.063/0.234)	0.52 (0.059/0.234)	0.28 (0.318/0.423)	0.42 (0.136/0.327)

*tmRT correlates with Behavioral Regulation index and Inhibition, Shifting, and Emotional Control scales. tmWM correlates with Global Executive Composite and marginally with Metacognition Index, Initiate Scale, Working Memory and Plan and Organize scales. Furthermore, tmWM correlates with Global Executive Composite. Format: rho (p/p-adj). Bold values indicate p < 0.05*.

## Discussion

We discovered that the novel threat modulation indices isolating the impact of threat-related distractor on different cognitive performance measures correlated with depression severity pre-ECT and executive functions in daily life post-ECT. To that end, these indices show potential as biomarkers of depression severity pre-ECT and cognitive outcome post-ECT. Such biomarkers might eventually prove beneficial for objective assessment of depression severity and for optimizing cognitive outcomes of depression treatments. Furthermore, in line with previous research, subjects' depression scores were reduced from pre-ECT level indicating severe depression to a level indicating mild depression post-ECT. We also discovered that executive functions and emotion regulation improved post-ECT, as indicated with both objective (Executive RT Test) and subjective (BRIEF-A questionnaire) measures.

While improved executive functions post-ECT have been previously reported (62), to our knowledge, this is the first study to show, in addition to objective improvement in executive functions in a testing environment also subjective improvement in daily life. In Executive RT Test, subjects' performance was more accurate post-ECT. Specifically, post-ECT subjects were more attentive and in better control of their responses, and post-ECT threat-related distractor interfered less with subjects' working memory performance or even improved it. In line with objective performance indicators, BRIEF-A scores reflecting the participants' subjective assessment of their executive functions in daily life indicated improved executive functions post-ECT. There was an improvement in their overall executive functions (Global Executive Composite Score), improvement in capability to control emotions and behavior (Behavioral Regulation Index), and improvement in practical problem-solving capability and working memory (Metacognition Index). The most notable improvement was in initiation of tasks, which is remarkable in daily functioning considering frequently debilitating lack of initiative in depression.

In large meta-analysis by Semkovska and McLoughin (62), impaired executive functions were initially observed during 0–3 days post-ECT, but pre-ECT level was recovered at 4–15 days, and improvements beyond the pre-ECT level were observed later. In the current study, executive functions were found to be improved earlier than in the meta-analysis, already 4–8 days after the completion of ECT. On the other hand, in the current study, reaction times were still prolonged at this point of time, while in the meta-analysis, speed of processing had resumed to pre-ECT level already 4–15 days post-ECT. Initial post-ECT slowing of processing speed is a well-known phenomenon (63) and may be linked with cerebral hypoperfusion (64). We speculate that traditional tests of executive functions are insensitive to subtle changes in executive functions (18, 19, 65). Executive RT Test can detect subtle improvement earlier and, on the other hand, depict slowed processing speed longer after ECT than traditional pen-and-paper tests with temporal resolution in the order of seconds as opposed to millisecond range in the Executive RT Test.

One could argue that instead of true improvement in the executive functions, the results merely reflect a change in speed accuracy tradeoff (66), since subjects were more accurate but had prolonged reaction times post-ECT. However, a slower processing speed was not detrimental in daily life tasks requiring executive functions, as results from BRIEF-A questionnaire indicated improved executive functions in daily life. One may consider a dissociation in the impact of ECT on cognitive functions, in which executive functions were improved but processing speed reduced in the acute early stage post-ECT. On the other hand, slowed post-ECT reaction time may not necessarily reflect impairment, but rather faster pre-ECT reaction times may reflect hasty or impulsive responding, especially in the context of threat-related stimuli. Indeed, pre-ECT threat-related distractors speeded up reaction times, and the greater the speed-up due to threat, the greater the depression severity. Threat-related stimuli have previously been shown to impair response inhibition in healthy subjects (30, 67), suggesting that hasty or impulsive responding due to threat is not limited to depression.

As expected, ECT caused changes in subjects' emotional reactivity and emotion–cognition interaction. Threat-related distractor interfered less and even improved subjects' working memory performance post-ECT compared to pre-ECT. Working memory is known to be vulnerable to interference (68–70), and the results suggest that working memory was more efficiently shielded from interference by threat-related distractor post-ECT. Likewise, there was a marginally significant change in tmRT, indicating that relative to neutral distractor, threat-related distractor speeded up RTs pre-ECT more than post-ECT. From the evolutionary point of view, it is plausible that the greater the perceived threat, the greater the impact on cognitive performance (71). Thus, it is possible that post-ECT subjects with mild depression perceived threatening distractors less threatening than pre-ECT when they suffered from more severe depression. However, as the perceived threat was not assessed in the current study, this is only a speculation.

Correlation analysis revealed further details about the emotional reactivity. When the association between the threat modulation index and depression severity was studied, pre-ECT tmRT, and tmWM were negatively associated with depression severity (Table 3A). Threat-related distractors speeded up RTs of more severely depressed subjects while slowing down RTs of less severely depressed subjects. The impact of emotional distractor on RTs of less severely depressed subjects resembles that of healthy individuals, as it has been shown that negative, task-irrelevant distractors impair healthy subjects' performance (26, 29).

Previous depression literature suggests that depressed individuals have hyperactive subcortical structures, such as the amygdala, and hypoactive cortical control structures, such as the dorsolateral prefrontal cortex (72–74). On the other hand, effective depression treatments have been shown to reduce amygdala reactivity to emotional stimuli (75). Hyperactive limbic structures along with hypoactive frontal circuits may contribute to a stronger than normal reaction to threat-related stimuli increasing arousal (76), which would facilitate faster RTs and better accuracy in more depressed individuals. Yet, another explanation for a negative distractor improving performance of more severely depressed subjects may relate to more impaired executive functions, slowed RTs, or lower baseline arousal level. Lower baseline arousal level leaves more room to increase the arousal level in contrast to less depressed subjects with closer-to-optimal cognitive performance and arousal levels, where increase in arousal may not improve performance any more, but rather impair it according to the Yerkes–Dodson Law (39, 77).

The impact of threat on performance in Executive RT Test reflected efficiency of executive functions in daily life as measured with BRIEF-A post-ECT. More specifically, the impact of threat on RTs reflected efficiency of emotion and behavior regulation, while the impact of threat on working memory reflected overall executive functions, metacognition, and efficiency of working memory. Post-ECT correlation analysis indicated that tmRT had a strong (*ρ* > 0.7) and tmWM moderate (*ρ* > 0.5) correlation with several BRIEF-A scales (Table 3B). Interestingly, the tmRT index was specifically associated with scales reflecting emotional and behavioral control and the tmWM index with scales reflecting metacognition and working memory. Moreover, tmWM index had a strong correlation (*ρ* = 0.72) with Global Executive Composite. Positive correlation between tmWM index and metacognition scales in BRIEF-A suggests that the lower the tmWM index, the less there are subjective challenges in metacognitive abilities in daily life. In other words, reduced interference or even enhancement of working memory due to threat reflects better metacognitive abilities. Negative correlation between tmRT and emotional and behavioral control scales suggests that the higher the tmRT index, the less challenges there are in behavior and emotion regulation in daily life. To that end, slowing of RTs with threat-related stimuli reflected better behavior and emotion regulation.

Similar to pre-ECT correlation with BDI, relatively more positive tmRT index indicates more “normal” reaction to threat-related distractor, now correlated with behavior and emotion regulation in daily life. Threat-related distractors compete for the same attentional resources with the task-relevant stimuli (78–80). Likewise, adaptive regulation of behavior and emotional responses compete for the same executive function resources required to carry out the task, interfering with task performance. The slowed RTs due to threat-related stimuli may reflect a cost on performance due to dual task situation where, in addition to the ongoing task, unexpected emotion regulation task adds up to response time. This contrasts with the situation where emotion regulation does not take place and responses may be hasty or impulsive. Threat may result in impulsive responding even in healthy subjects (30). Thus, faster RTs in context of threat-related stimuli in severely depressed subjects may be due to lacking emotion regulation along with automatic emotional arousal effect.

Despite several strengths, there are limitations in this study. This was not a clinical intervention study but rather a small experimental study, where we assessed depression and cognitive outcomes pre- and post-ECT in a treatment-resistant MDD group who received ECT for clinical reasons. Depression severity, executive functions, and emotion–executive function interaction were measured with BDI, MADRS, BRIEF-A, and performance in Executive-RT test. Thus, some of the limitations are inherently linked to small sample size and the study design, where we lack the control group and randomization of an intervention due to ethical reasons. To that end, the results should be considered preliminary, and conclusions need to be taken with appropriate caution. Especially conclusion regarding improved cognitive functions should be considered in the context of well-known ECT-mediated cognitive impairments linked particularly to memory functions (81).

The within-subject approach used in the study could have confounding factors related to the repeated testing, most notably learning effect instead of a genuine improvement of executive functions due to ECT. However, we have previously shown the reliability of repeated assessment of executive functions with Executive RT Test when initial learning is accounted for (49), and those approaches were strictly applied in this study.

Regarding limitations of the threat modulation indices, tmRT and tmWM, as depression biomarkers, there was a lack of correlation with MADRS pre-ECT and with BDI and MADRS post-ECT. While both BDI and MADRS assess depression severity, they differ in a critical way, as BDI is a self-report measure of the depression severity unlike clinician-rated MADRS. The fact that MADRS lacks a structured interview may affect reliability and consequently contribute to lack of correlation with the threat modulation indices pre-ECT. Lack of correlation post-ECT may relate either to the lack of sensitivity of these indices in mild depression, lack of adequate variance in mild depression scores post-ECT, or to a small sample size. Another reason for lack of correlation of threat modulation indices with depression metrics post-ECT could relate to habituation to threat-related stimuli. We did not evaluate subjective perception of the threat nor unpleasantness of threat-related stimuli in this study. The threat-related stimuli were task-irrelevant small black line drawings of spiders presented in the middle of a visually significantly more salient task-relevant stimulus, large and colorful traffic light. Thus, selective attention was not voluntarily geared to emotional distractors. Distractor stimuli were also presented very briefly, only for 150 ms, and many of the subjects were not even aware of distractors while focusing on cognitively challenging task. It is likely that biologically relevant prototypical stimuli engage rapid and evolutionarily hard-wired pathways directly from the thalamus to the amygdala, making the effect of threat stimuli rapid and rather robust (82). The effect of threat on cognitive functions do not necessarily require subjective perception.

In the future, to further investigate and develop biomarkers of depression based on the impact of threat on cognitive performance, it will be critical to study habituation to threat stimuli and weather potential habituation depends on efficiency of frontal control circuits or depression severity. However, the fact that threat-related stimuli continued to have an impact on cognitive performance post-ECT and that tmRT correlated with several behavior regulation related BRIEF-A scales and tmWM with several metacognition related BRIEF-A indices suggests that subjects did not habituate to these stimuli, at least to the extent where threat would not have any impact on their performance.

Another potential confounding factor in the study is the possibility that the study group was not a representative sample of the typical patients receiving ECT for refractory MDD. There are some challenges in the recruitment of subjects with severe MDD to participate in a study, which requires significant cognitive effort. Volunteering as a subject in a research study requires cognitive energy and initiation abilities, which are frequently compromised in MDD. Many subjects withdrew their consent during the first test session due to lack of sufficient energy for carrying out the study task. Thus, there may have been an unintentional bias in the study group favoring subjects with adequate level of mental energy over those with severe lack of energy.

In summary, these preliminary results from a small study with aforementioned limitations suggest alleviated depression and improved executive functions and emotion regulation 4–8 days post-ECT. Subjects were less impulsive and more accurate in computerized test of executive functions and experienced improved executive functions in daily life. Furthermore, their reactions to emotional distractors were normalized, and their working memory better shielded from emotional interference. Threat modulation index correlated with depression severity pre-ECT and with BRIEF-A questionnaire scores post-ECT. The fact that threat modulation indices reflect the level of depression in more severe depression and level of executive functions and emotion regulation in milder depression may highlight how intricately intertwined these phenomena are. In milder depression, threat-modulation biomarker might reflect the resilience or susceptibility to reoccurrence of depression. This, however, requires further studies to be confirmed.

The current study presented novel threat modulation indices that may serve as biomarkers for depression severity pre-ECT in more severely depressed population and as indicators of emotion regulation and efficiency of executive functions in milder depression post-ECT. In addition to potentially contributing to improved diagnostic tools, such biomarkers have the potential and applicability in scientific endeavors aiming at better understanding the role of executive functions and emotion regulationin depression.

## Data Availability Statement

Restrictions apply to the datasets. The datasets for this article are not publicly available because the written consent forms signed by the participants did not provide information concerning public distribution of data.

## Ethics Statement

The studies involving human participants were reviewed and approved by Tampere University Hospital Ethical board. The patients/participants provided their written informed consent to participate in this study.

## Author Contributions

JP was the main author of the manuscript, participated in data collection, data analysis, interpretation of the results, and authoring of the manuscript. KJ was responsible for the recruitment of subjects, ECT as part of subjects' clinical treatment and participated in interpreting the results, and authoring the manuscript. PH was responsible for data collection and participated in authoring the manuscript. JC participated in interpretation of the results and authoring of the manuscript. KH was responsible for the experimental design, invented the novel biomarkers based on threat-modulation of cognitive performance, supervised data collection and data analysis, participated in the interpretation of the results, and authoring of the manuscript. All authors contributed to the article and approved the submitted version.

## Conflict of Interest

The authors declare that the research was conducted in the absence of any commercial or financial relationships that could be construed as a potential conflict of interest.
